# Impact of an oral care intervention protocol on oral health outcomes in head and neck cancer patients undergoing radiation or chemoradiation therapy

**DOI:** 10.1186/s12903-025-05877-8

**Published:** 2025-04-11

**Authors:** Radhika R. Pai, Sourjya Banerjee, Linu Sara George, Anice George, Ravikiran Ongole

**Affiliations:** 1 Fundamentals of Nursing Department, Manipal College of Nursing, Manipal Academy of Higher Education, Manipal, India; 2 Department of Radiation Oncology, Kasturba Hospital, Mangalore, India; 3 Child Health Nursing Department, Manipal College of Nursing, Manipal Academy of Higher Education, Manipal, India; 4 Department of Oral Medicine and Radiology, Manipal College of Dental Sciences, Manipal Academy of Higher Education, Mangalore, India

**Keywords:** Oral health outcomes, Head and neck cancer patients, Radiation therapy, Chemoradiation, Oral care intervention protocol

## Abstract

**Background:**

Head and neck cancer patients receiving chemotherapy and radiation therapy may experience a notable and frequently sudden decline in their oral health. These alterations include oral mucositis that develops during and shortly after treatment, candida infections, trouble speaking, difficulty eating, bleeding gums, and tissue fibrosis.

**Materials and methods:**

This study aimed to determine the effectiveness of oral care intervention protocol (OCIP) on oral health and oral complications. The experimental group received a structured oral care protocol, and the control group received oral care as per the standard of care of the study setting. These patients were observed every week for up to 6 weeks until the completion of radiation therapy/chemoradiation. An experimental design using a randomized controlled trial was adopted for the study. After providing informed consent, the data were collected from 80 head and neck cancer patients.

**Results:**

The maximum number of patients, i.e., 42.5% in the experimental group and 32.5% in the control group, were diagnosed with tongue cancer. Most of the participants, i.e., 57.5% in the experimental group and 67.5% in the control group, received chemoradiation as the treatment plan. Among all the oral complications, the median days to develop mucositis (*p* =.015), swallowing difficulty (*p* =.009), and chewing difficulty (*p* =.032) were significantly different from those of the control, indicating that the intervention was effective. As treatment progressed over the weeks, the severity of the oral problems increased in both groups (*p* =.001). Compared with routine care, oral care intervention improved oral health scores among cancer patients receiving head and neck radiation therapy/chemoradiation [F (401.982), *p* =.001].

**Conclusion:**

These data suggest that the OCIP is clinically helpful in maintaining overall oral health among cancer patients receiving head and neck radiation/chemoradiation. The OCIP effectively delays the incidence of oral complications arising from head and neck radiation therapy/chemotherapy but does not prevent them. The findings of this study can also contribute to providing evidence for the use of an oral care kit, including all evidence-based interventions for patients receiving head and neck radiation/chemoradiation.

## Introduction

Cancer patients receiving chemotherapy and head and neck radiation therapy may experience a notable sudden decline in their oral health [1–4]. The acute effects of RT include mucositis, thickened secretions, mucosal infections, pain, and sensory disruptions. The long-term chronic effects of head and neck RT include tissue fibrosis, salivary gland dysfunction, increased susceptibility to mucosal infections, neuropathic pain, sensory disorders and increased susceptibility to dental caries and periodontal disease [2, 4–7]. Oral problems are distressing to patients, which may impact their physical, psychological, and social well-being and demonstrate a high degree of intensity [8–11]. Oral problems significantly affect global health status and health-related quality of life, especially among those receiving head and neck radiation [12–14].

To enhance oral care for patients undergoing chemotherapy, a diverse team of specialists from various fields, including medicine, dentistry, nursing, nutrition, physical therapy, and counseling, is essential [15]. The Multinational Association of Supportive Care in Cancer/International Society for Oral Oncology’s expert panel advocates (Level of Evidence III) for the implementation of combined oral care protocols to prevent oral mucositis during chemotherapy, head and neck radiation therapy, and hematopoietic stem cell transplantation [16]. Management of these treatment-induced complications necessitates collaboration among medical oncologists, radiation oncologists, and dentists via a multidisciplinary approach to examine the oral cavity and detect early signs and symptoms of cancer treatment-related oral changes [17]. A thorough approach to oral care for cancer patients was supported by the 2007 MASCC/ISOO guidelines, which placed a strong emphasis on the value of dental evaluation prior to starting treatment. The guidelines suggested an interdisciplinary approach to oral care, the use of validated tools for clinical evaluation and patient self-reporting, and the establishment of a systematic and structured oral care routine based on the data currently available. Using a soft toothbrush, replacing it frequently, flossing, and using uninteresting rinses and moisturizers were all part of this routine [18].

Regular oral hygiene did not address complications seen post-radiation and chemotherapy, such as mucositis, candida infection, taste loss, xerostomia, swallowing difficulty, and nutritional imbalance. It is very essential to implement tailored and individualized management strategies to address these symptoms effectively [6, 19]. Organizations must also follow a particular oral care protocol that will aid in treating oral symptoms. Using a safe and effective systematic oral care protocol offers patients a proper way of performing oral care, such as cleaning, nourishing, guarding, and increasing salivation. Maintaining good oral hygiene can lessen the effects of oral problems [20–23]. The literature recommends implementing a uniform oral care protocol for mucositis management reduces mucositis occurrence, duration, and severity and decreases the worldwide negative impact of mucositis [22, 24–26].

The literature supports the importance of oral care among cancer patients to reduce the adverse effects of cancer treatment [1, 4, 27, 28]. The implementation of oral preventive measures may contribute to improving the prognosis of squamous cell carcinoma (SCC) patients by reducing the negative impact of oral complications [29]. Despite the variability of oral care protocols, the overall results revealed a significant reduction in oral complications when these protocols were adhered to properly. However, these reported studies did not investigate the clinical effectiveness of a simple oral care protocol for assessing oral complications among cancer patients. The present study aimed to assess the clinical effects of implementing an oral care protocol among cancer patients receiving chemotherapy and radiation therapy.

### Materials and methods

The study was conducted between November 2015 and March 2020 in oncology-related wards of the tertiary care center in Mangaluru, southern India. Inclusion criteria for the study were patients who were in any stage of cancer, who were undergoing chemoradiation, who were admitted to radiation oncology and special wards, who were scheduled for radiation therapy to the head and neck region, who were receiving only radiation therapy or postoperative radiation therapy, who were receiving at least 75% of both parotids in the radiation field, who received the radiation agent Linac (linear accelerator), who received an average radiation dose of 60–70 Gy and who were willing to participate in the study. The exclusion criteria for the study was patients with cancers other than those affecting the oropharyngeal region.

This randomized control trial (RCT) used purposive sampling to select the participants. The sample size for the study was calculated for the primary outcome variable, oral health, via hypothesis testing of the two population means formula [30]. where Z1-α/2 = 1.96 (level of significance at 5%), Z(1-β) = 0.84 (power at 80%), $\:{\upsigma\:}2\:$= 4.95 (variance based on the pilot study), and d2 = 2.25 (clinically significant difference in oral health status). Thirty-four participants were included in each group. Considering a 20% attrition rate, an additional six participants were included. Hence, the total sample size required for the intervention and control groups was 80.

The intervention and control groups were selected from different wards to avoid sample contamination. Based on a random sampling number, an allocation sequence was generated. According to a computer-generated randomization list, participants were allocated to the intervention or control group (research randomizer available at www.randomizer.org). Blocks were established to ensure equal numbers of subjects in each treatment group. Eighty head and neck cancer patients were recruited for the present study. Four blocks, with a fixed block size of 10, were set aside for each group to allocate a 1:1 ratio to maintain balance within the groups. Allocation concealment was achieved via sequentially numbered opaque sealed envelopes (SNOSE). Concealment was ensured by numbering the envelopes in advance, and during the intervention assignment, the envelopes were opened sequentially only after participant details were written on the appropriate envelope.

With the help of random allocation software (RAS), a statistician performed sequence generation. A colleague, with the help of the randomization list, prepared SNOSE. The intervention group received an oral care protocol, an oral care kit, and patient education materials, which were prepacked in a ziplock sachet and numbered for each patient according to the randomization schedule. The subject expert enrolled the participants, and the nurse coordinator, who was not involved in the care of the recruited patients, was assigned to open the envelopes and allocate the patients to interventions. The allocation sequence was concealed from the guide/subject experts. The subject expert in this study was responsible for diagnosing the oral complications, and this information was identified by the researcher from the records based on the treatment written in patient records for each complication.

Patients were aware of their allocation to the study group. The researcher was responsible for providing oral care kits and education materials and explaining them to the patients, training staff nurses in the ward, implementing the oral care protocol, and documenting oral care. The researcher and trained staff nurses were aware of the allocation. The outcome assessor (subject expert) was blinded to the allocation and the assessment of outcomes. Hence, this was an outcome-blinded study.

**Fig. 1:**
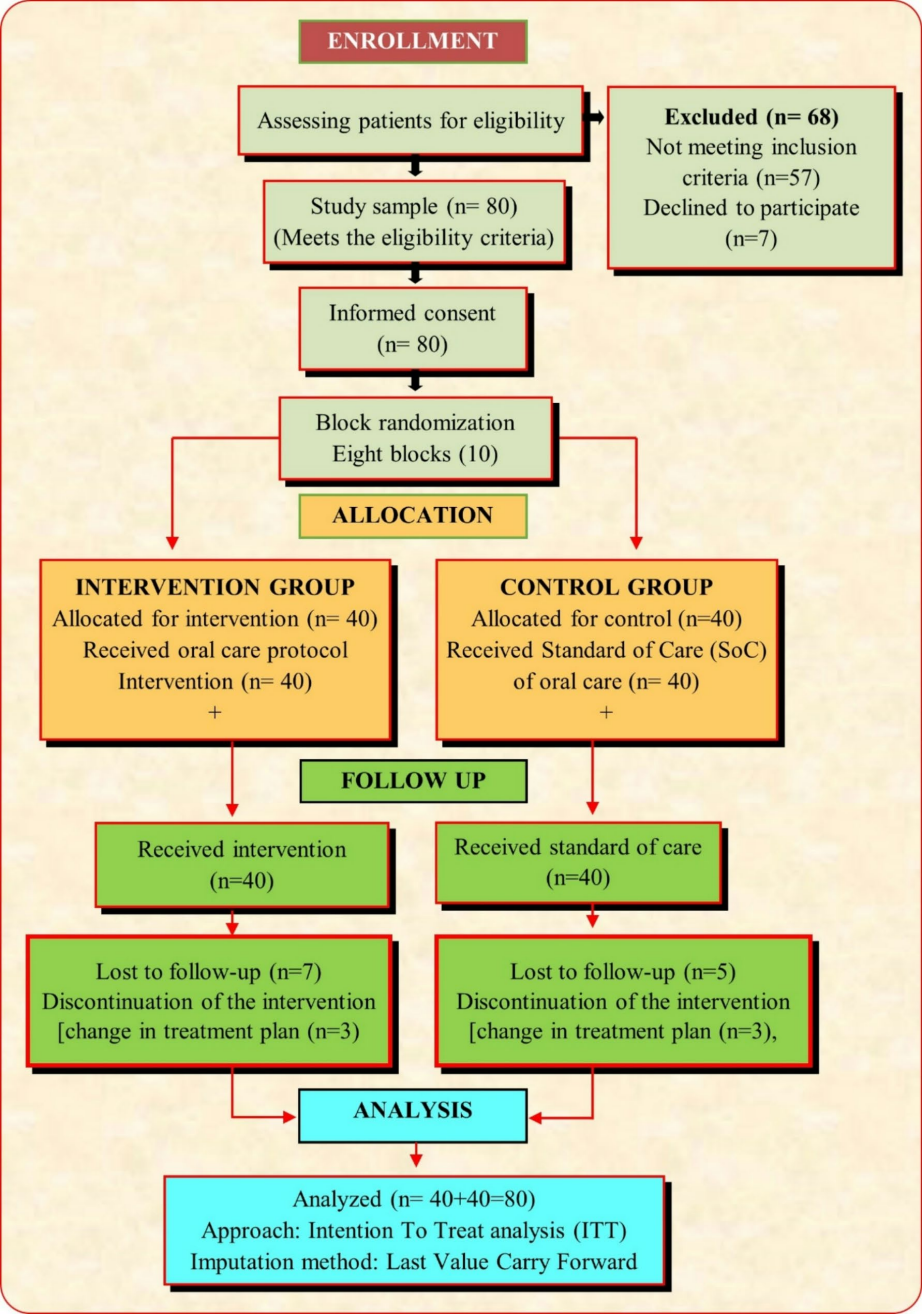
Consolidated Standards of Reporting Trials (CONSORT) flow chart

The study’s progress in terms of enrolment, allocation, follow-up, and subsequent data analysis is summarized in the CONSORT flow diagram (Fig. 1).

### Tools

The demographic data of the patients were collected during the patient’s first visit after providing informed consent from both the intervention and control groups. Clinical tools were developed to measure the clinical details of the participants, which consisted of type/location of cancer, stage of cancer, type of treatment, chemotherapy drug, radiation dose, and body mass index (BMI), which were obtained from the records.

The oral health and activity assessment tool assesses functional aspects and oral activities, including pain, taste, infection, saliva, lips, teeth, tongue, and mucous membrane. Self-oral care, speaking, chewing, and swallowing were among the activities. Every element was assigned a score according to its appearance. The final score was thirty. A score of 10 denoted good oral health, a score of 11–20 indicated moderate risk, and a score of 21–30 indicated high risk of oral problems. The patients were assessed daily after introducing the oral care kit until their cancer treatment course was completed via a checklist. The expected duration for completing the treatment was 1½ months, which meant a minimum of 6 assessments during their stay. The reliability of the oral health assessment tool was established via an interrater reliability technique. The researcher and a nurse independently observed oral health among 20 patients, and the calculated reliability coefficient Cohen’s kappa coefficient was (*r* =.87), which showed agreement among the raters.

The WHO oral mucositis grading scale is commonly used in clinics to evaluate oral mucositis [31]. It is graded from 0 to 4. This tool was classified as Grade 0 = no symptoms; Grade 1 = soreness and erythema; Grade 2 = erythema, ulcers, and patients can swallow a solid diet; Grade 3 = ulcers, extensive erythema, patients cannot swallow a solid diet; and Grade 4 = mucositis to the extent that alimentation is not possible. The reliability of the oral mucositis grading scale was established with an interrater reliability technique. The researcher and a nurse independently observed oral health among 15 patients, and the calculated reliability coefficient was (*r* =.94), which showed agreement between the raters.

The oral complication incidence checklist consisted of 9 oral complications and their days of occurrence after the initiation of radiation therapy/chemoradiation. The complications assessed were oral mucositis, taste loss, swallowing difficulty, speaking difficulty, oral candida infection, xerostomia, bleeding gums, and nutritional imbalance. The treating physician recorded the treatment details on the care sheet when these complications were identified. Information was collected from patient records where the treating oncologist had written the prescription.

### Intervention details

#### Oral care intervention protocol (OCIP): intervention group

The intervention group received this study’s oral care on the basis of the oral care intervention protocol (OCIP). The researcher administered an oral care kit and education materials and trained the patients. The trained staff nurses in the ward helped implement oral care per the protocol and documented oral care.

The oral care kit used in this study consisted of an ultrasoft bristle toothbrush, fluoridated toothpaste, oral rinses (salt, baking soda), chewy tubes, a mirror, a pen torch, a denture brush, a denture container, an ice cube box, a water bottle, and patient educational material (leaflet on oral care during treatment and after discharge, on oral complications, menu plan during the treatment).

The patient education materials were validated by seven experts from oral medicine and radiology, radiation oncology, medical oncology, and medical-surgical nursing. Patient education material was translated into the local language, Kannada, and retranslated to the English version of the education leaflet and compared with the translated English version. This was done to check for any deviation from the original content due to translation errors by giving the data to a language expert for verification.

#### Control group

The control group received routine oral care per the hospital’s standard of care (SOC).

Both the intervention and control groups were given regular monitoring, treatment, and follow-up services delivered by physicians, nurses, and other healthcare team members. (Table 1)

**Table 1:** Schematic representation of the research design

Groups	Pretest	Intervention	Posttest (6 weeks)
Intervention ^R^	-	**X** **Oral care intervention protocol (OCIP)**	0_1_ Oral Health Oral complications
Control ^R^	-	**Standard of care of oral care**	

**Keywords**: R random assignment/randomization, O observation, 0_1_ Posttest

### Data collection process

An intervention was launched in two stages following the creation of a systematic oral hygiene strategy. Here, the patients received oral care through the research intervention, which included providing denture care, reading patient education materials, chewing the chewed tube before eating, brushing with an ultrasoft toothbrush and fluoridated toothpaste, and rinsing the mouth with soda bicarbonate. The patient underwent this oral care procedure four times a day. In addition to these interventions, the patient received a detailed meal plan that specified what foods one should eat and what she should avoid while undergoing therapy, as well as instructions on how much water one should drink each day, two to three liters, to improve hydration status. This intervention phase included a one-year follow-up period for the patients once they completed their radiation therapy/chemoradiation by auditing the patient records.

Informed consent was obtained from the participants, and their demographic data were collected. The primary outcome measures in this study were the incidence of oral complications, oral mucositis, and oral health status. The occurrence of oral complications was collected from patient records and patients’ verbalizations of the oral problems. The WHO mucositis grading scale was used to assess oral mucositis. Oral health was assessed with a structured checklist. These tools were used once every week until the patient completed the prescribed dose of radiation therapy/chemoradiation. A documentation audit of patient records concerning the implementation of oral care and oral complications occurred during the study period.

## Results

In the present study, most participants were included, i.e., 51.2% in the intervention group and 52.5% in the 56-year-old and older age groups. Most patients were males, with 92.5% in the intervention group and 87.5% in the control group. Most patients, i.e., 40% in the intervention group and 37.5% in the control group, reported having a primary education. Seventeen (56.7%) patients in the intervention group and 13 (43.3%) in the control group had tongue cancer. (Table 2)

**Table 2:** Sociodemographic characteristics of the patients *N* = 80

Variables	Intervention (*n* = 40)		Control (*n* = 40)		*p* value
	f	%	f	%	
**Age in years**					
18–55	18	48.6	19	47.5	1.0
56 and >	22	51.2	21	52.5	
**Gender**					
Male	37	92.5	35	87.5	0.712
Female	3	7.5	5	12.5	
**Education**					
Illiterate	15	37.5	13	32.5	
Primary education	16	40	15	37.5	0.706
Higher Secondary	7	17.5	11	27.5	
Graduation	2	5	1	2.5	
**Type and location of the cancer**					
Tongue	17	42.5	13	32.5	
Buccal mucosa	3	7.5	7	17.5	
Larynx	1	2.5	1	2.5	
Supraglottis	3	7.5	4	10	
Hypopharynx	6	15	5	12.5	0.706
Oropharynx	7	17.5	6	15	
Tonsil	1	2.5	0	0	
Maxilla	0	0	1	2.5	
Pyriform fossa	1	2.5	2	5	
Glottis	1	2.5	0	0	
Nasopharynx	0	0	1	2.5	
**BMI**					
Malnourished	8	20	8	20	
Underweight	10	25	15	37.5	0.521
Normal	19	47.5	16	40	
Overweight	3	7.5	1	2.5	

*P* >.05

Overall, 23 (57.5%) head and neck cancer patients in the intervention group and 27 (57.4%) in the control group received chemoradiation therapy. Among patients who received chemoradiation therapy, most were treated with cytoplatin and kemocarb as chemotherapy agents. On average, most patients in the intervention (29 [72.5%]) and control (34 [85%]) groups received 70 Gy of radiation. With respect to the TNM classification, 20 (50%) and 15 (37.5%) of the head and neck cancer patients were in the T4 subgroup, 21 (52.5%) and 25 (62.5%) were in the N2 subgroup, and 34 (85%) and 32 (80%) were in the Mx subgroup in the intervention and control groups, respectively. Most of the patients, 19 (47.5%) in the intervention group and 17 (42.5%) in the control group, had stage IV cancer at diagnosis. (Table 3)

**Table 3:** Description of the treatment and staging details *N* = 80

Variables	Intervention (*n*=40)				Control (*n*=40)				*p* value	
	f			%	F			%		
**Type of treatment**										
Radiation therapy	17			42.5	13			32.5	0.173	
Chemoradiation	23			57.5	27			67.5		
**Chemoradiation**										
**Agent**										
Cytoplatin	9			39.13	10			25		
Cisplatin	2			8.69	0			0	0.290	
Kemocarb	8			34.78	10			25		
Gefitinib	1			4.34	3			7.5		
Chemoplat	2			8.69	4			10		
Unicarb	1			4.34	0			0		
**Radiation therapy**										
**Dose in Grays (Gy)**										
60	4			10	3			7.5	0.343	
66	7			17.5	3			7.5		
70	29			72.5	34			85		
**TNM classification**										
**Tumor**										0.148
T1		3	7.5			0	0			
T2		7	17.5			9	22.5			
T3		10	25			16	40			
T4		20	50			15	37.5			
**Node**										0.569
N0		12	30			12	30			
N1		3	7.5			1	2.5			
N2		21	52.5			25	62.5			
N3		4	10			2	5			
**Metastasis**										0.770
Mx		34	85			32	80			
M0		6	15			8	20			
**Stage of cancer**										0.687
I Early localized		1	2.5			1	2.5			
II Early locally advanced		8	20			8	20			
III Late locally advanced		12	30			14	35			
IV Metastasized		19	47.5			17	42.5			

*P* >.05

### Effect of the oral care intervention protocol (OCIP) on oral health

**Fig. 2:**
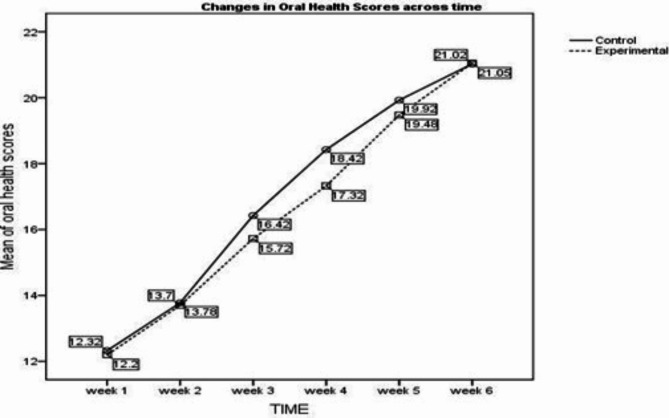
Profile plot showing changes in oral health across six weeks

The results of the present study revealed that, compared with the control group, improvement in oral health was not statistically significant among patients in the intervention group [F = (0.596), *p* =.442]. However, the interaction effect (time × group) was not significant [F = (1.636), *p* =.175]. This result indicated that the group changed over time and differed across the groups. (Fig. 2)

Repeated-measures ANOVA revealed a significant effect of time (within the group) [F (401.982), *p* =.001]. This can be interpreted as patients in both groups improving their oral health over time. Compared with routine care, oral care intervention improved oral health among cancer patients receiving head and neck radiation therapy/chemoradiation. (Table 4)

**Table 4:** Repeated-measures ANOVA on the effectiveness of the OCIP on oral health *N* = 80

Variable	Groups		F	df	*P* value	ƞ *p*^2^
	Intervention (*n* = 40)	Time	401.982	5,390	0.001	0.837
Oral Health	Control (*n* = 40)	Group Time X Group	0.596 1.636	1,78 5,390	0.442 0.175	0.008 0.021

Note: The score ranges from 10–30. Time refers to within-group effects, group refers to between-group effects, and time X refers to interaction effects. SD – standard deviation = F ratio, df = degrees of freedom, p – level of significance (< 0.05), ƞ p2 = partial eta squared (effect size)

A lower score on the Oral Health Assessment Checklist reflected better oral health, whereas a higher score indicated structural and functional changes in the oral cavity. This profile plot also indicated better oral health in the intervention group than in the control group. More definite differences in the oral health scores were observed between 3 and 5 weeks, indicating that as the intervention progressed, improvements in oral health were observed in the intervention group compared with the control group. Compared with routine care, oral care intervention improved oral health among cancer patients receiving head and neck radiation therapy/chemoradiation.

### Effect of the oral care intervention protocol (OCIP) on oral structure and function

As treatment progressed, the severity of the structural changes and functional activities increased in both groups over the week, and they also increased in both groups.

**Table 5:** Comparison of mean days of onset of symptoms related to oral complications *N* = 80

Oral complications	group	Mean (SD)	F	Mean difference	CI (difference)	Independent sample t test (pdf)	*p* value
Mucositis	Intervention control	17.12 (7.58) 11.02 (4.22)	14.031	6.1	3.36–48.83	4.444 (78)	< 0.001 *
Chewing difficulty	Intervention control	17.12 (7.26) 13.88 (6.00)	1.642	3.25	0.28–6.21	2.181 (78)	0.032*
Speaking difficulty	Intervention control	17.55(7.00) 16.05(5.93)	0.500	1.5	-1.38-4.38	1.304(78)	0.482
Candida Infection	Intervention control	19.68(9.51) 18.92(6.04)	1.841	1.8	-2.37-5.97	0.858(78)	0.179
Xerostomia	Intervention control	20.72(10.47) 17.58(8.14)	0.802	1.62	-2.34-5.59	0.815(78)	0.417
Bleeding gums	Intervention control	19.68(9.51) 18.92(6.04)	1.826	0.75	-2.79-4.29	0.421(78)	0.675
Nutritional imbalance	Intervention control	20.05(6.33) 21.32(9.01)	1.312	1.27	-2.19-4.74	0.732(78)	0.466

*P <.05

Table 5 shows that, on average, it took nearly 17 days for the onset of mucositis symptoms in the experimental group compared with the control group, where it occurred in 11 days. The mean difference of 6 days between the intervention and control groups concerning mucositis incidence was statistically significant (*p* <.001). On average, 17 days were needed for the onset of symptoms of chewing difficulty in the experimental group compared with the control group, which occurred after 14 days. The mean difference of 3 days between the intervention and control groups concerning the incidence of chewing difficulty was statistically significant (*p* =.032). These findings indicate that the treatment effectively delayed the incidence of mucositis and chewing difficulty in the intervention group.

However, complications such as speaking difficulty occurred at an average of 18 days for the onset of symptoms in the intervention group compared with those in the control group at 16 days (*p* =.482); on average, it took nearly 20 days for the onset of oral candida infection symptoms in the intervention group compared with that in the control group at 19 days (*p* =.179); on average, it took nearly 21 days for the onset of symptoms related to xerostomia in the intervention group compared with that in the control group at 18 days (*p* =.417); on average, it took nearly 20 days for the onset of symptom-related bleeding gums in the intervention group compared with that in the control group at 19 days (*p* =.675). On average, it took nearly 20 days were needed for the onset of nutritional imbalance (inability to eat orally) in the intervention group compared with the control group at 21 days (*p* =.466). These complications were not statistically significant, indicating that the intervention was effective for these oral complications.

**Table 6:** Comparison of the median days of onset of symptoms related to oral complications Mann‒Whitney U test *N* = 80

Oral complications	Group	Median	Mean(SD)	(Q1, Q3)	Mann‒Whitney ‘U’	*P* value
Taste loss	Intervention	12.5	12.62(6.96)	(8,16)	492	0.828
	Control	10.5	12.38(6.26)	(9,16.25)		
Swallowing difficulty	Intervention	12	12.72(6.52)	(9,16)	527	0.009*
	Control	16	16.95(7.48)	(11,21)		

*P <.05

The median number of days to onset of swallowing difficulty symptoms was four days greater in the intervention group than in the control group, indicating that the intervention effectively reduced swallowing difficulty (*p* =.009). Compared with those in the control group, complications such as taste loss were not significantly different (*p* =.828), indicating that the intervention was ineffective in reducing the severity of taste loss in the intervention group. (Table 6)

## Discussion

The present study used an oral care kit, including all evidence-based interventions for patients receiving head and neck radiation/chemoradiation. In the literature, various studies have used different procedures and components to provide oral care for cancer patients. A similar approach is taken up by an expert committee that reported the use of empirically tested best clinical practices as standards for practitioners’ routine oral care of cancer patients [22]. Another study too reported the critical facets of oral care, assessment, and treatment, including various oral care strategies at hospitals, hospices, and home settings, are integral components of an oral care module developed to address oral care in cancer care in London, UK [32].

Literature highlights the development of oral care protocols incorporating various oral care agents and their impact on oral health. Similarly, an oral care protocol was developed by Sieracki et al. in Pittsburgh, USA, using a patient-centered approach guided by a nurse who used a new oral care regimen, including flossing, rinsing the mouth with regular saline, and examination of the oral cavity [33]. Oral hygiene procedures and the routine use of a mouth rinse solution were reported as the basis of a suitable oral care protocol. This further promoted strict adherence to the oral care protocol, which can help prevent oral complications [34]. An ongoing education update for oral care for head and neck cancer symptom management in Iowa City, USA, suggested the use of an oral kit, which included an ultrasoft toothbrush, dental floss, salt, baking soda packets for oral rinsing, Biotene-based toothpaste, a denture cup and other small cups for mixing. One study reported a decrease in the effects of oral mucositis during and after radiation treatment [35]. Comparable results have been documented in a study that showed the effectiveness of consistent professional oral health care, including dental cleaning, scaling, brushing directions, and lifestyle guidance, lowering the risk of oral mucositis in patients receiving chemotherapy for breast cancer in Japan [35].

Findings from this study suggest that the mean difference observed over 6 weeks between the intervention and control groups indicated that the intervention was effective in delaying the incidence of mucositis (*p* ≤.001) and chewing difficulty (*p* ≤.032), which was statistically significant. The median number of days to swallowing difficulty was greater in the intervention group than in the control group, indicating that the intervention effectively reduced swallowing difficulty (*p* =.009). An intervention study reported an increased incidence of mucositis in the self-care group compared with the professional oral care intervention group in a study where an oral care protocol was introduced [36]. Related conclusions have been drawn in a study where the standard care alone group experienced mucositis symptoms more frequently than did the standard care plus plain ice and standard care plus flavoured ice groups [37]. An oral care protocol was introduced in Michigan, USA, to promote early detection and management of stomatitis, reporting an increased number of cases after implementation, i.e., 11 compared with the retrospective records of 5 patients. Additionally, nurses perceived a benefit for their patients and reported good nursing practices after the intervention [38]. Equivalent results have been stated in a study where significant improvement in oral health conditions between the initial assessment and the two longitudinal assessments (*p* <.05) was reported in a study conducted in Brazil, indicating that the oral preventive care program was effective for plaque control and reduced gingival inflammation [29].

The current study reported a significant effect of oral care protocol on mucositis, swallowing difficulty, and speaking difficulty but not on other related oral complications. Corresponding evidence has been found in a similar study where a rigorous oral care regimen alone cannot reduce the incidence of severe oral mucositis in patients with HNC receiving chemoradiotherapy. Regular dental care programs and the use of best practices may indirectly help enhance treatment compliance by lowering the risk of mucositis and related infections [3, 39]. Another study reported comparable outcomes where a specific oral hygiene regimen reduces the development of mucositis and is negatively correlated with opportunistic infections [40].

However, there are limitations inherent to the present study. The sample was representative of cancer patients who met the inclusion criteria mentioned in the study. The researchers could not observe the practice of the oral care intervention, as it was difficult for the researcher to be present throughout the intervention. Only verbal reports of the continuity of oral care by the patients and their relatives were collected during each visit concerning adherence to the protocol. In addition, the researchers and participants were not blinded to the intervention.

## Conclusion

Oral complications are the most common post-radiation therapy/chemotherapy-associated complications and may cause significant morbidity and mortality. A combined effort by multiple professionals, such as medical oncologists, radiation oncologists, oral medicine specialists, and oncology nurses, can result in the development of simple yet effective patient care interventions, including specific oral care protocols and oral care kit interventions for the oral care of cancer patients receiving head and neck radiation/chemoradiation. An OCIP effectively delays the incidence of oral complications such as oral mucositis, chewing difficulty, and swallowing difficulty but does not prevent these complications. However, the incidence of complications such as oral candida infection, speaking difficulty, bleeding gums, xerostomia, taste loss, and nutritional imbalance did not significantly differ, indicating that oral care intervention was ineffective for these oral complications. Significant mean and median differences in the incidence of oral complications were detected between the intervention and control groups. These data suggest that the OCIP is clinically valuable for maintaining overall oral health among cancer patients receiving head and neck radiation/chemoradiation. The findings of this study can also contribute to justifying the use of an oral care kit, including all evidence-based interventions for patients receiving head and neck radiation/chemoradiation, which is potentially useful for future comparisons.

## Data Availability

Upon reasonable request and with permission of the Manipal Academy of Higher Education, the data will be made available by the corresponding author.

## Abbreviations

OCIP: Oral care intervention protocol
RT: Radiation Therapy
CT: Chemotherapy
